# Graphene Supported Rhodium Nanoparticles for Enhanced Electrocatalytic Hydrogen Evolution Reaction

**DOI:** 10.1038/s41598-019-53501-x

**Published:** 2019-11-19

**Authors:** Ameerunisha Begum, Moumita Bose, Golam Moula

**Affiliations:** 1 Department of Chemistry, Faculty of Science, Jamia Hamdard University, New Delhi, 110062 India; 2 Department of Chemistry, University of Calcutta, Acharya Prafulla Chandra Road, Calcutta, 700009 West Bengal India

**Keywords:** Chemistry, Nanoscience and technology

## Abstract

Current research on catalysts for proton exchange membrane fuel cells (PEMFC) is based on obtaining higher catalytic activity than platinum particle catalysts on porous carbon. In search of a more sustainable catalyst other than platinum for the catalytic conversion of water to hydrogen gas, a series of nanoparticles of transition metals *viz*., Rh, Co, Fe, Pt and their composites with functionalized graphene such as RhNPs@f-graphene, CoNPs@f-graphene, PtNPs@f-graphene were synthesized and characterized by SEM and TEM techniques. The SEM analysis indicates that the texture of RhNPs@f-graphene resemble the dispersion of water droplets on lotus leaf. TEM analysis indicates that RhNPs of <10 nm diameter are dispersed on the surface of f-graphene. The air-stable NPs and nanocomposites were used as electrocatalyts for conversion of acidic water to hydrogen gas. The composite RhNPs@f-graphene catalyses hydrogen gas evolution from water containing p-toluene sulphonic acid (p-TsOH) at an onset reduction potential, E_p_, −0.117 V which is less than that of PtNPs@f-graphene (E_p_, −0.380 V) under identical experimental conditions whereas the onset potential of CoNPs@f-graphene was at E_p_, −0.97 V and the FeNPs@f-graphene displayed onset potential at E_p_, −1.58 V. The pure rhodium nanoparticles, RhNPs also electrocatalyse at E_p_, −0.186 V compared with that of PtNPs at E_p_, −0.36 V and that of CoNPs at E_p_, −0.98 V. The electrocatalytic experiments also indicate that the RhNPs and RhNPs@f-graphene are stable, durable and they can be recycled in several catalytic experiments after washing with water and drying. The results indicate that RhNPs and RhNPs@f-graphene are better nanoelectrocatalysts than PtNPs and the reduction potentials were much higher in other transition metal nanoparticles. The mechanism could involve a hydridic species, Rh-H^−^ followed by interaction with protons to form hydrogen gas.

## Introduction

The design of a complex that can catalyze electrochemical hydrogen activation at lower potentials than platinum electrode under ambient conditions is a challenge. The presence of the dimetallic iron and heterodimetallic iron-nickel centers at the active sites of the hydrogenases has invoked to search for a non-platinum catalyst material for the purpose^1–8^. Hydrogenases are enzymes that catalyze the interconversion of H_2_ and its constituents, two protons and two electrons as shown in Eq. (1).

1 $$2{{\rm{H}}}^{+}+2{{\rm{e}}}^{-}\to {{\rm{H}}}_{2}$$

**Figure 1:**
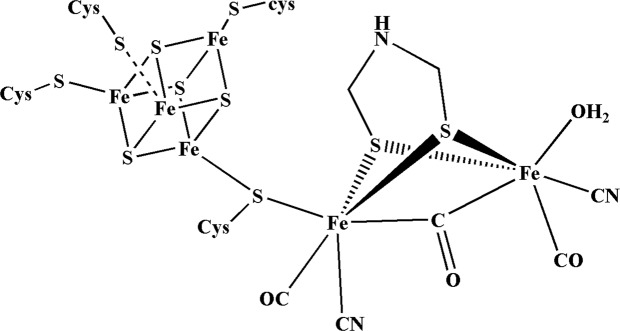
H-cluster, the active site. Structure of the Hydrogen-cluster in the H_2_ evolving bacterium *Clostridium pasteurianum*^1^.

The three known classes of hydrogenases, [NiFe]-, [FeFe]- and FeS-cluster free hydrogenases contain iron at their active sites which are coordinated by thiolates, CO, CN^−^ or a light sensitive cofactor^2^. The [Fe_4_(μ_3_–S_4_)] cubane type sub-cluster obtains electrons from pyruvate oxidation and transfer those electrons to the [FeFe] site which utilizes them and form hydrogen gas in an unique reaction mechanism. The mechanism involves the replacement of the labile water ligand shown in Fig. 1 by a proton followed by reductive elimination supported by a free cysteine residue (Cys^299^) at the vicinity of the [FeFe] cluster^1^.

Although nature uses the potential redox active element “iron” in this active site, the hydrogen gas electrode is based on platinum metal. Electrocatalytic H_2_ gas generation is the conversion of electricity to H_2_ gas in the presence of a catalyst that can performance-wise replace the platinum electrode which functions at E_p_, −0.413 V at a pH value of 7.0^8^. Iron-containing electrochemical catalysts that are known to date include mononuclear iron(II) complexes, [Fe^II^(S_2_C_6_H_4_)(CO)_2_(PMe_3_)_2_], [Fe^II^(S_2_C_6_H_2_Cl_2_)(CO)_2_(PMe_3_)_2_], an FeS cluster with Fe_cubane_(μ-SR)Fe_subsite_ and [Fe_2_(CN)(CO)_4_H(PMe_3_)(S_2_C_3_H_6_)] which function at high reduction potentials, *E*p ≈ −1.0 V^9–16^. However, several electrochemical catalysts that contain other transition metals are known, e.g. carbon nanotube supported [Ni(PPh_2_NPh_2_)_2_(CH_3_CN)][BF_4_]_2_, (CpMoμ-S)_2_S_2_CH_2_, cobalt macrocyclic glyoxime/tetraimine complexes and a nickel(II) dithiolene complex which function at considerably lower reduction potentials. Several other electrochemical hydrogen evolution catalysts have also been reported including metalloporphyrins, low-valent transition metal complexes forming hydrides upon reaction with acids, mononuclear iron(II) complexes, cobalt-dithiolene complexes and a nickel complex [Ni(P^Ph^_2_N^Ph^)_2_][BF_4_]_2_ (P^Ph^_2_N^Ph^ = 1,3,6-triphenyl-1-aza-3,6-diphosphacycloheptane) which electrocatalyze H_2_ production with high turnover frequencies but at significantly high reduction potentials E_p_ > −1.1 V^17–24^. Various kinds of heterogeneous non-precious metal based electrocatalysts for hydrogen evolution reaction (HER) and oxygen reduction reaction (ORR) including metal sulfides, selenides, carbides, nitrides, phosphides, three dimensional porous carbon nanostructures and heteroatom doped nanocarbons have been reviewed^25–27^. Multilayer thin films of metal nanocrystals and graphene quantum dots have been prepared by layer-by-layer assembly approach and these composites demonstrate efficient and versatile electrocatalytic performances such as reduction of aromatic nitro compounds, methanol oxidation and water splitting^28^. Plasmonic TiO_2_ NRs@Ag@GQDs ternary heterostructures which have been prepared by a layer-by-layer assembly strategy combined with an *in situ* light irradiation display an enhanced photoelectrochemical water splitting performance^29^. Pd-CdS nanowire heterostructures display remarkable photocatalytic reduction of nitroarenes and photocatalytic hydrogen production under visible light irradiation^30^. Our research focused towards the electrochemical hydrogen evolution resulted in a mononuclear iron(III) dithiolene of severely distorted square pyramidal geometry and a nickel(II)-sulfur based radical ligand complex that catalyze electrochemical hydrogen gas evolution at lower potentials in CH_3_CN. In these cases, along with metals, the dithiolene ligands are also potentially redox active forming sulfur based radicals^31–36^.

It has been demonstrated that Pt_3_Ni(111) surface is 90-fold more active for ORR than the current platinum catalyst on porous carbon used in PEMFC^37^. Core-shelled TiC@TiO_2_ has been shown to be a promising catalyst support for proton exchange membrane fuel cells (PEMFCs). TiC is thermally stable with low solubility in sulfuric acid and high electronic conductivity. Both these materials are used as supports for platinum and platinum–palladium alloy catalysts (Pt/TiC, Pt_3_Pd/TiC and Pt_3_Pd/TiC@TiO_2_) and their catalytic activity toward ORR are much higher than those for Pt/TiC^38^. Earth abundant transition metal nanocatalysts that include Mn, Fe, Co, Ni and Cu and early transition metals such as Ti, V, Cr, Zr, Nb and their nanocomposites for reduction of various aromatic compounds have been reported^39^. Synthesis of different types of graphene-based composite photocatalysts and their applications in reduction of CO_2_, nitroarenes, methanol oxidation, elimination of pollutants and photochemical water splitting have been reviewed^40^. Au-Pd nanoalloys supported on graphene (Au-Pd/GR) have been reported which display higher photocatalytic performance than the monometallic, GR supported nanoparticles towards degradation of dye, rhodamine B (RhB)^41^. Basic principles of photocatalytic water splitting, engineering strategies for photocatalysts optimization, and promising photocatalytic materials for water splitting have been recently reviewed^42^. Various metal oxides, metal non-oxides and non-metal catalysts for oxygen evolution reaction (OER) have also been recently reviewed^43^. There are several reports on the catalytic activities of rhodium nanoparticles (RhNPs) and rhodium nanoparticles on polymer, graphene and carbon nanotube matrices for the reduction of aromatic compounds, amino boranes as a means of H_2_ storage. But there are no reports on the application of RhNPs and graphene supported RhNPs (RhNP@f-graphene) as electrocatalysts for the generation of hydrogen gas from water. Here in we report preparation, characterisation and electrocatalytic properties of rhodium nanoparticles (RhNPs) and graphene supported rhodium nanoparticles (RhNPs@f-graphene) which display better electrocatalytic performance than the platinum nanoparticles (PtNPs) and graphene supported platinum nanoparticles (PtNPs@f-graphene) under similar experimental conditions.

## Results and Discussion

### Preparation of nanoparticles of rhodium (RhNPs), platinum (PtNPs) and cobalt (CoNPs) (1–3)

**Figure 2:**
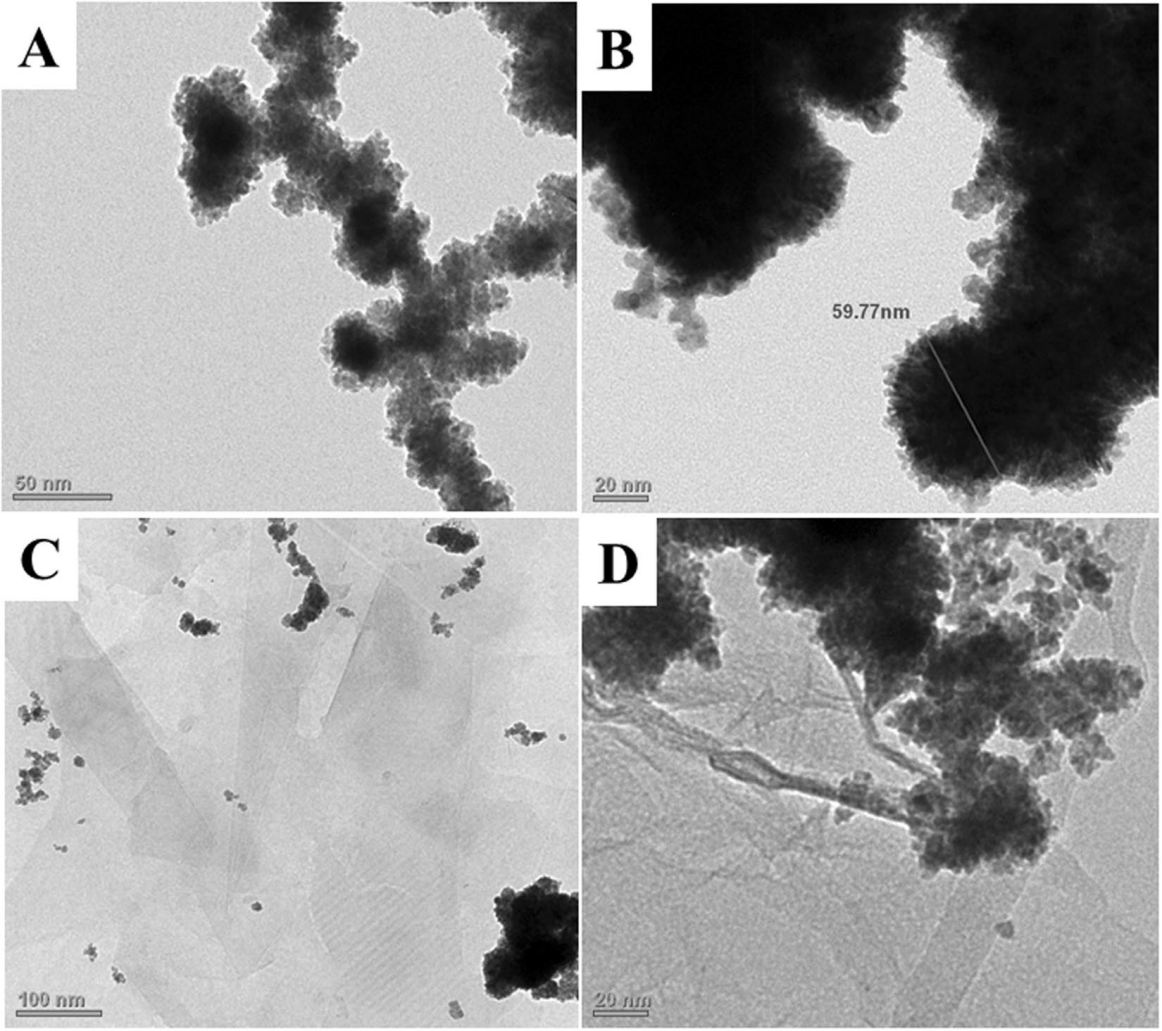
TEM images of RhNPs and RhNPs@f-graphene. (**A**) RhNPs at low magnification; (**B**) RhNPs at high magnification; (**C**) RhNPs@f-graphene; (**D**) RhNPs@rGO.

**Figure 3:**
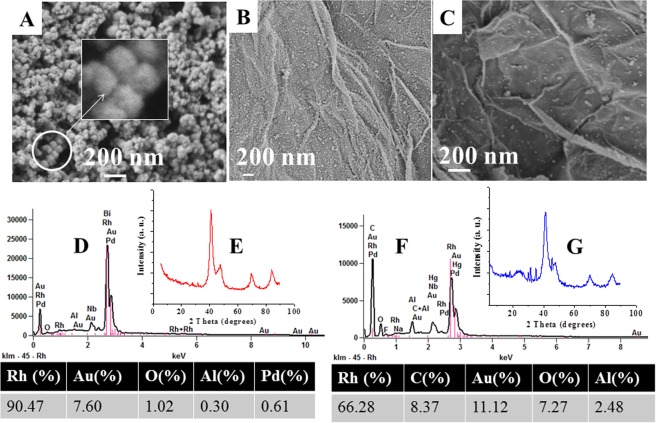
SEM analysis of RhNPs and RhNPs@f-graphene. (**A**) SEM image of RhNPs with inbox showing aggregation of nanoparticles; (**B**) SEM image of RhNPs@f-graphene at low magnification; (**C**) SEM image of RhNPs@f-graphene at higher magnification; (**D**) EDX analytical result of RhNPs; (**E**) Powder XRD spectrum of RhNPs; (**F**) EDX analytical result of RhNPs@f-graphene; (**G**) Powder XRD spectrum of RhNPs@f-graphene.

**Figure 4:**
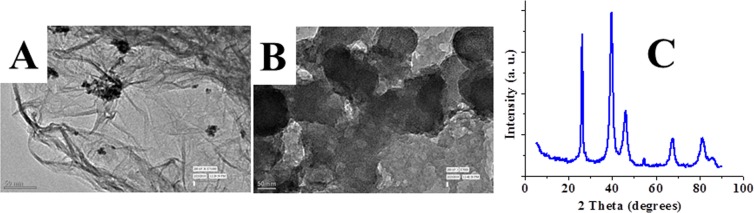
TEM images of the composites. (**A**) TEM image of PtNPs@f-graphene; (**B**) TEM image of CoNPs@f-graphene; (**C**) Powder XRD spectrum of PtNPs@f-graphene.

The detailed synthetic procedures for the preparation of the three nanoparticles RhNPs, PtNPs and CoNPs, (**1**–**3**) are given in methods. The metal halides were reduced by NaBH_4_ in water at laboratory temperature (30 °C) under high dilution conditions. The black colored particles which precipitated out immediately were sonicated, centrifuged and isolated after 12 h. The transition metal nanoparticles (TMNP, **1**–**3**) were insoluble in water, they were sonicated in water and deposited on to polymer coated carbon grids and aluminum stubs for transmission electron microscopic (TEM) and scanning electron microscopic (SEM) analysis respectively. The TEM analytical results of the rhodium nanoparticles are shown in Fig. 2A and a higher magnification image of RhNP is diaplayed in Fig. 2B. The RhNPs of <10 nm diameter are found to agglomerate to form nanostructures of 60 nm diameter as shown in Fig. 2B. The SEM images of RhNPs on aluminium stubs are displayed in Fig. 3A which further confirms the agglomeration of RhNPs to nanostructures of 60 nm diameter. The nanoparticles were confirmed to be pure rhodium nanoparticles by EDX analysis as shown in Fig. 3D. Powder XRD spectrum of RhNPs was measured in the range of 2θ from 5° to 90°. As shown in Fig. 3E, a sharp peak was observed at 2θ, 41.2° and broad, weak peaks were displayed at 2θ, 47.4°, 69.5° and 83.8°. The TEM images of the PtNPs and CoNPs are shown in Fig. 4. The PtNPs were polydispersed and are of 50 nm and 30 nm diameters as shown Fig. 4A and the CoNPs were found to group into spherical structures of 89 nm diameter as shown in Fig. 4B. The SEM image of the CoNPs also indicate the agglomeration of the particles as shown in Supplementary Information.

### Preparation of composites, TMNPs@f-graphene

The functionalized graphene was obtained from graphitic powder by following a previously reported procedure^31^. Graphitic powder was sonicated in tetrahydrofuran (*thf*) for 1 h followed by centrifugation. The supernatant *thf* layer was decanted and the residue was washed with acetone and dried thoroughly which was heated under reflux in a mixture of H_2_SO_4_ and HNO_3_. The composites of transition metal nanoparticles and functionalized graphene were obtained by sonication of both in acetonitrile. The solvent was evaporated and the residue was washed with acetone and then finally dried. The composites were characterized by TEM and SEM methods. The TEM images of the RhNP@f-graphene are given in Fig. 2C,D which clearly indicate the presence of graphene sheets and RhNPs. The TEM images further indicate that RhNPs of <10 nm diameter are dispersed on graphene sheets. The aggregated RhNPs are found to segregate upon formation of composite with f-graphene as shown in Fig. 2C. The SEM images of the RhNP@f-graphene composite shown in Fig. 3B,C indicate that the dispersion of RhNPs on graphene sheets resemble dispersion of water droplets on lotus leaves. The SEM images of the RhNP@f-graphene further confirm that the size of the rhodium nanoparticles is about 11–13 nm. The EDX analysis of the composite show that the percentage of rhodium is 66.2% and carbon content is 8.3% as shown in Fig. 3F. The powder XRD spectrum of RhNPs@-graphene in the range of 2θ from 5° to 90° is displayed in Fig. 3G which shows an additional weak and broad peak at 2θ, 24.6° along with the peaks at 2θ, 41.2°, 47.6°, 69.9°, 84.3°. The weak and broad signal at 2θ, 24.6° in RhNPs@f-graphene confirms the integration of RhNPs with f-graphene^44^. The TEM images of the platinum nanoparticles (PtNPs) and cobalt nanoparticles (CoNPs) are displayed in Supplementary Information whereas the TEM images of the f-graphene composites of PtNP and CoNP are displayed in Fig. 4A,B respectively. The PtNPs are polydispersed and are of 30–60 nm in diameter whereas the CoNPs agglomerated to form nanostructures of 80 nm in diameter. The TEM images of the f- graphene composites of PtNPs and CoNPs indicate that the metal nanoparticles are immobilized on the functionalized graphene and agglomerated as shown in Fig. 4A,B. The powder XRD spectrum of the composite, PtNPs@f-graphene is shown in Fig. 4C. The composite PtNPs@f-graphene displayed sharp peaks at 2θ, 39.7°, 46.2°, 67.5° and 81.1° which are the standard powder XRD signals for platinum nanoparticles along with a signal at 2θ, 26.3° indicating the integration of platinum nanoparticles with f-graphene^45^.

### Electrocatalytic studies

**Figure 5:**
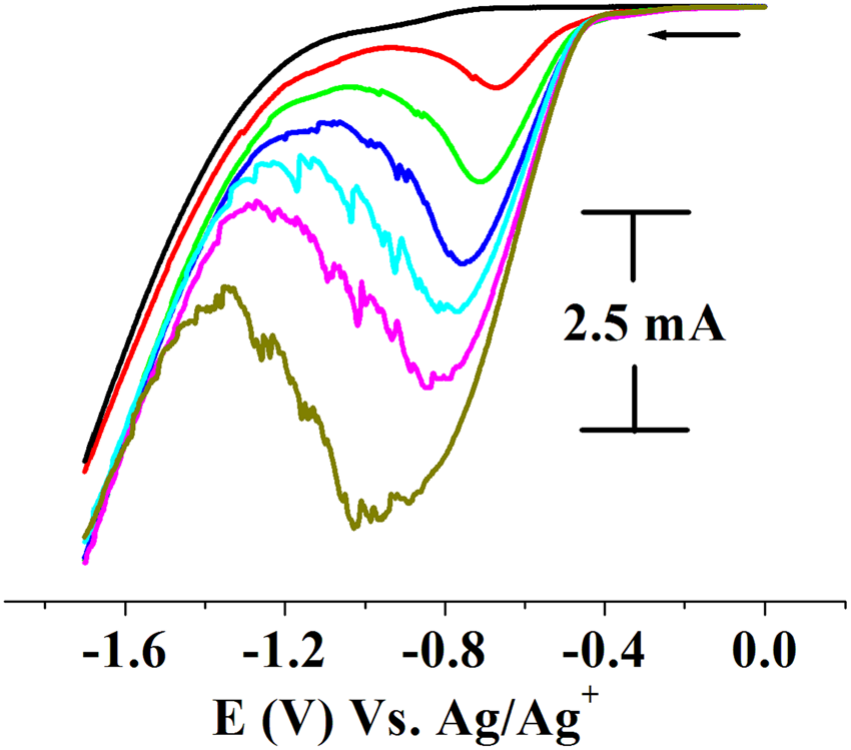
Electrocatalysis by RhNPs. Cyclic voltammograms of RhNPs as a function of increasing concentrations of added *p*-TsOH (red to olive green) in water. (0.5 M *p*-TsOH, 0.2 mL each in water). Displaying only forward reduction waves for clarity; scan rate of 100 mVs^−1^ (Supporting electrolyte, KNO_3_/H_2_O (0.2 M), GCE working, Pt wire auxillary and Ag/AgCl reference electrodes).

**Figure 6:**
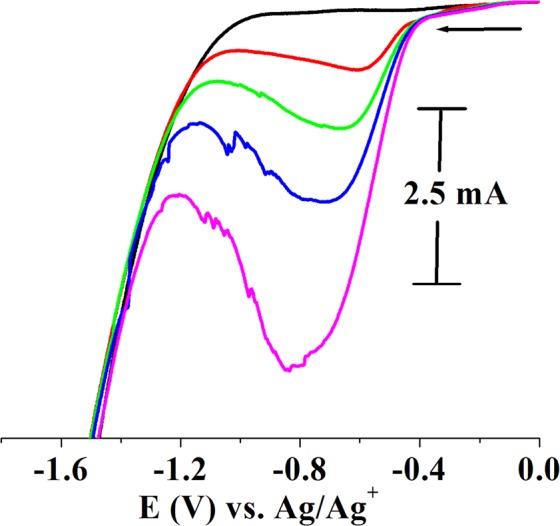
H_2_ evolution from acidic water by RhNPs@f-graphene. Cyclic voltammograms of RhNPs@f-graphene as a function of increasing concentrations of added *p*-TsOH (red to magenta) in water. (0.5 M *p*-TsOH, 0.2 mL each in water). Displaying only forward reduction waves for clarity; scan rate of 100 mVs^−1^ (Supporting electrolyte, KNO_3_/H_2_O (0.2 M), GCE working, Pt wire auxillary and Ag/AgCl reference electrodes).

**Figure 7:**
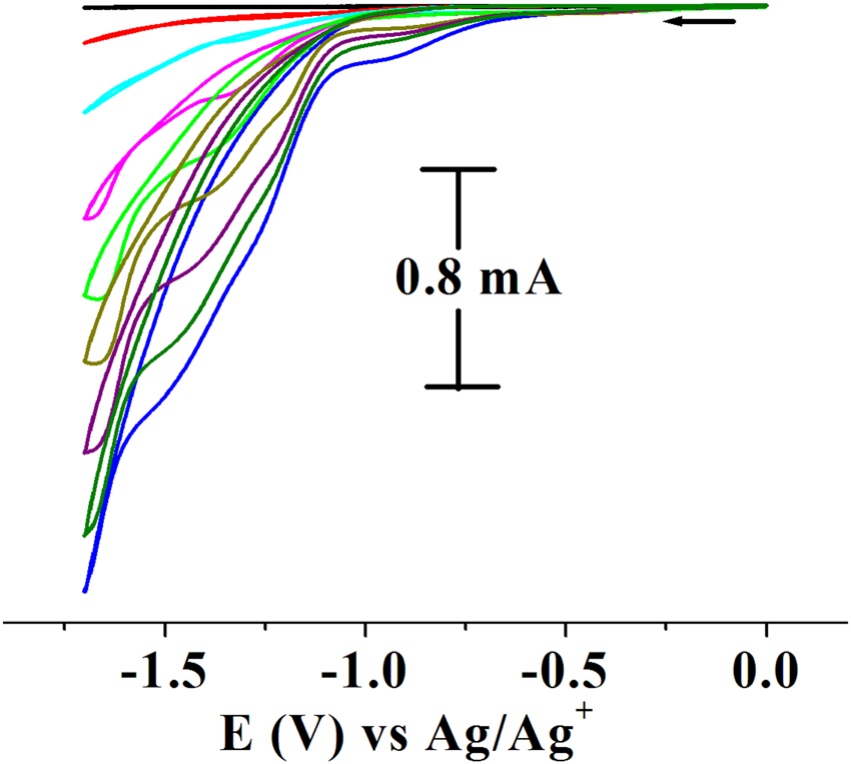
H_2_ evolution from acidic water by f-graphene in the absence of electrocatalysts. Cyclic voltammograms (black to blue) of f-graphene as a function of increasing concentrations of added *p*-TsOH in water. (0.5 M *p*-TsOH, 0.2 mL each in water). scan rate of 100 mVs^−1^ (Supporting electrolyte, KNO_3_/H_2_O (0.2 M), GCE working, Pt wire auxillary and Ag/AgCl reference electrodes).

The transition metal nanoparticles and their f-graphene composites were tested for electrocatalytic efficiency and hydrogen gas generation by cyclic voltammetry. The electrocatalysis was conducted in acidic water containing *p*-TsOH. Glassy carbon working, platinum wire auxillary and Ag/AgCl reference electrodes were used. In a typical experiment, nanoparticles (0.02 g) were taken in distilled water (8 ml) in an electrochemical cell, with potassium nitrate (0.1 mM) as a supporting electrolyte. Aqueous solutions of *p*-TsOH were added to the electrochemical cell and the cyclic voltammograms were recorded as a function of increasing concentrations of *p*-TsOH. The results of electrocatalytic experiments using RhNPs and RhNP@f-graphene as the catalysts are displayed in Figs 5 and 6 respectively. Pure RhNPs did not show any peak (Fig. 5, black). Upon adding aqueous solutions of *p*-TsOH (0.2 ml of 50 mM) a reduction signal was observed at E_p_, −0.67 V (Fig. 5) whereas the RhNP@f-graphene displayed the reduction signal at E_p_, −0.601 V (Fig. 6). The reduction onset potentials were observed at E_p_, −0.186 V and −0.117 V for RhNPs and RhNP@f-graphene respectively. Compared with the pure RhNPs, the reduction potential of the composite RhNP@f-graphene was shifted by 60 mV towards E_p_, 0.00 V. Additions of p-TsOH to the cyclic voltammetric cell resulted in increasing currents as shown in Figs 5 and 6. The RhNPs and RhNPs@f-graphene consumed 0.8 g of p-TsOH and the current exceeded 10 mA. Gas bubbles sticking to the glassy carbon working electrode were observed during electrolysis. The gas bubbles were analyzed and confirmed to be hydrogen gas by head space analysis^32^. A mixture of RhNPs@graphene (0.02 g) and *p*-TsOH (0.8 g, ~4 mmol) in water with KNO_3_ (0.2 M) as supporting electrolyte was purged with nitrogen gas for 15 min. A 2 ml syringe was inserted into the electrochemical cell and the argon gas supply inlet was closed. The reaction mixture was subjected to controlled potential electrolysis at −0.6 V. The gas bubbles formed at the glassy carbon working electrode surface were tapped off to the surface and the gas over the solution was taken into a syringe which was analysed by gas chromatography and confirmed to be H_2_ gas. Cyclic voltammograms of functionalized graphene without electrocatalysts were recorded as a function of addition of aqueous solutions of p-TsOH and the results are displayed in Fig. 7. As a function of increasing concentrations of added p-TsOH (black to blue) under similar experimental conditions, only graphene electrocatalyzes proton reduction at a potential of E_p_, −1.7 V but the onset potentials were observed at E_p_, −1.1 V. The reduction of pure *p*-TsOH in water in the absence of RhNPs or RhNPs@f-graphene was observed at a higher negative potential, E_p_, −1.4 V vs. Ag/Ag^+^.

### Mechanism of proton activation

**Figure 8:**
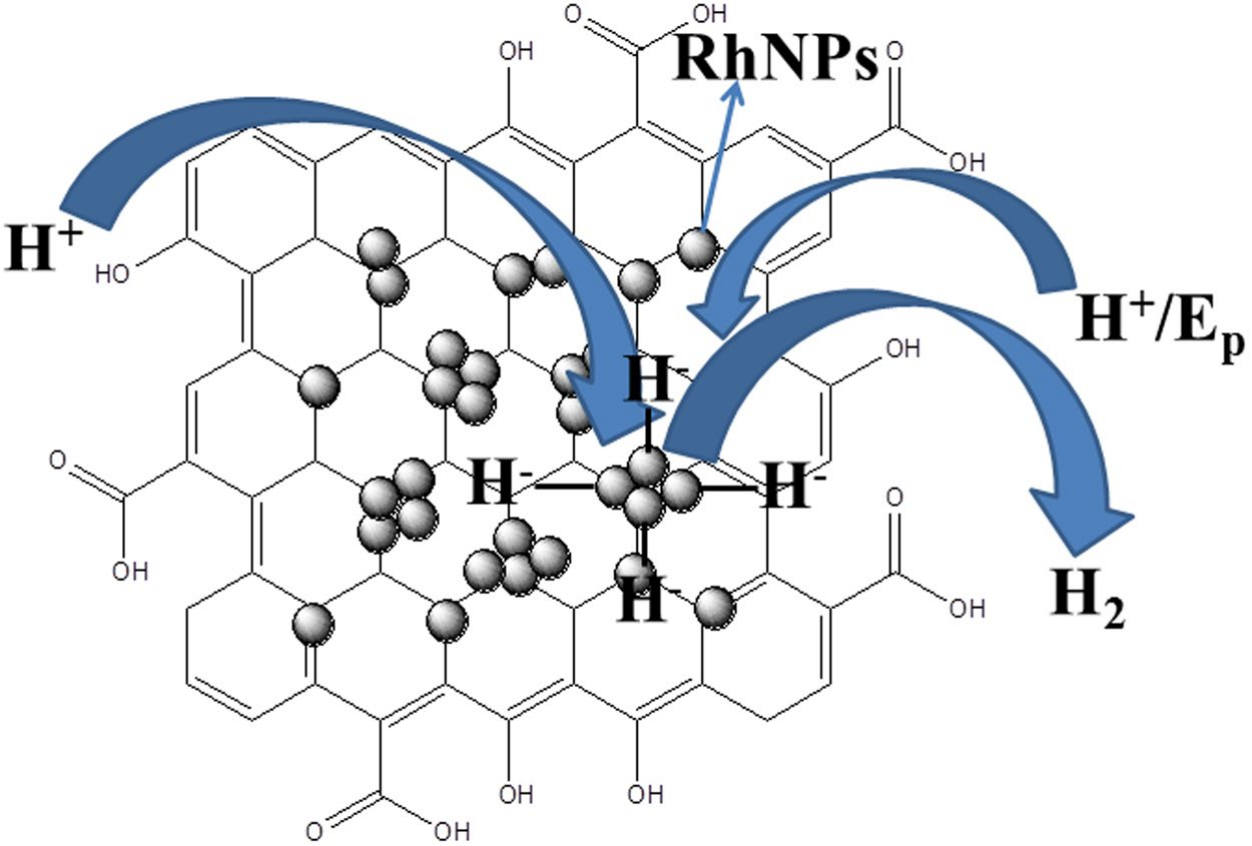
Mechanism of reduction of protons to hydrogen gas on RhNPs@f-graphene. Display of RhNPs on a functionalized graphene layer which take up protons and convert to hydrogen gas.

The SEM analytical results of the composite, RhNPs@f-graphene (Fig. 3C) indicate that the RhNPs are dispersed over f-graphene layers similar to the dispersion of water droplets on a lotus leaf. The interaction between the RhNPs and functionalized graphene could be π-bonding interaction or dihapto type (η^2^-type) of boding and protons could also bind to rhodium leading to the formation of Rh-H^+^ species. This could be reduced upon application of electrode potential to a hydridic species, Rh-H^−^ as shown in Fig. 8. Structures of rhodium-hydride complexes coordinated to fullerenes (C_60_) in an η^2^-mode viz., (η^2^-C_60_)Rh(CO)H(PPh_3_)_2_ have been reviewed^46^. This Rh-H^−^ species could interact with further protons and could form hydrogen gas. In the process, rhodium nanoparticles remain unaffected and provide site for fixing protons which in other words, an adsorption medium. The rhodium metal nanoparticles could play an important role in bringing down the reduction potential, E_p_, because the reduction of protons and evolution of hydrogen gas takes place at higher reduction potentials when CoNPs (E_p_, −0.98 V) and FeNPs (E_p_, −1.59 V) were used as electrocatalysts. This might be due to the ease of formation and optimum stability of Rh-H^−^ species.

### Recycling and durability of RhNPs and RhNPs@f-graphene

After the cyclic voltammetric experiments, the RhNPs and the RhNPs@f-graphene were filtered from the reaction mixture and were analysed by SEM and TEM techniques. The results obtained in the case of RhNPs are shown in Supplementary Information which indicate the presence of mixtures of large crystals along with the RhNPs. The large crystals could be KNO_3_ supporting electrolyte and p-toluene sulfonate used in the cyclic voltammetric experiment. The electrocatalysts, RhNPs and RhNPs@f-graphene were recycled as follows: After the cyclic voltammetric experiment, the RhNPs and RhNPs@f-graphene were filtered and were sonicated in water (50 mL) for 30 min. Then they were centrifuged and the water layer was decanted. The process was repeated for 6 times in order to get rid of KNO_3_ and *p*-toluene sulfonate and the recycled and purified RhNPs and RhNPs@f-graphene were reused as the electrocatalysts. The recycled RhNPs were found to electrocatalyze further generation of hydrogen gas by addition of *p*-TsOH (0.8 g) and the current again exceeded 10 mA. This proves that the RhNPs and RhNPs@f-graphene have excellent durability and electrocatalytic efficiency.

### Comparison of electrocatalytic efficiency of RhNPs@f-graphene

**Figure 9:**
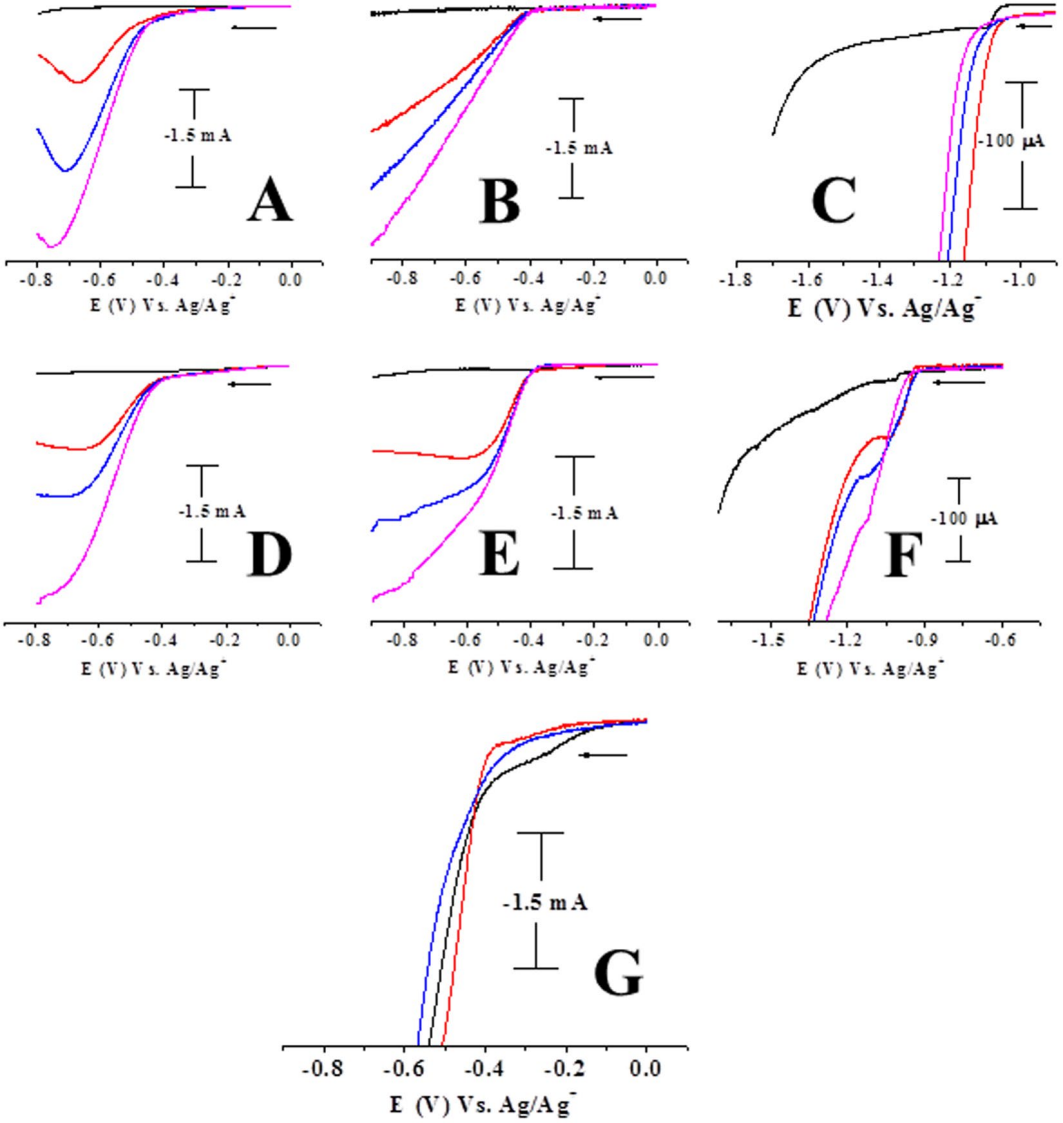
Comparison of the onset potentials and electrocatalytic efficiency of RhNPs@f-graphene with other transition metal@f-graphene nanocomposites. Cyclic voltammograms of (**A**) RhNPs: (**B**) PtNPs; (**C**) CoNPs; (**D**) RhNPs@f-graphene; (**E**) PtNPs@f-graphene; (**F**) CoNPs@f-graphene as a function of increasing concentrations of added *p*-TsOH in water. (0.5 M *p*-TsOH, 0.2 mL each in water); (**G**) Comparison of reduction on-set potentials. black, onset potential for RhNPs@f-graphene, red, on-set potential for PtNPs@-graphene, Blue, onset potential for RhNPs. Displaying only forward reduction waves for clarity; scan rate of 100 mVs^−1^ (Supporting electrolyte, KNO_3_/H_2_O (0.2 M), GCE working, Pt wire auxillary and Ag/AgCl reference electrodes).

In order to compare the catalytic efficiency of RhNPs and RhNP@f-graphene with that of Platinum electrodes under identical experimental conditions, electrocatalytic experiments were conducted using PtNPs and PtNPs@f-graphene. Electrocatalytic experiments were also conducted using CoNPs and CoNPs@f-graphene as electrocatalysts. The cyclic voltammetric results using PtNPs, PtNP@f-graphene, CoNPs, CoNP@f-graphene as electrocatalysts and as a function of increasing concentrations of three additions of p-TSOH are shown in Fig. 9. In these experiments the onset potential which is the lowest potential at which the reduction current starts to flow in the electrochemical system was monitored. The PtNPs displayed the onset potential at E_p_, −0.367 V (Fig. 9B) where as CoNPs displayed the onset potential at _Ep_, −0.98 V (Fig. 9C). The composite PtNP@f-graphene displayed the onset potential at E_p_, −0.38 V (Fig. 9E) and the composite, CoNP@f-graphene displayed the onset potential at E_p_, −0.968 V (Fig. 9F). But the onset potentials for RhNPs and RhNPs@f-graphene are very low at E_p_, −0.186 V (Fig. 9A) and E_p_, −0.117 V (Fig. 9D) respectively. The onset potentials for the hydrogen evolution from acidic water by RhNPs@f-graphene, PtNPs@f-graphene and pure RhNPs are compared in Fig. 9G. The cyclic voltammetric profile obtained on using RhNPs@f-graphene as electrocatalyst is displayed in black, that for PtNPs@f-graphene in red and that for the pure RhNPs in blue. These onset potentials clearly indicate the higher catalytic performance of RhNPs and its composite, RhNP@f-graphene than even PtNPs and PtNP@f-graphene under identical experimental conditions. The RhNPs and RhNP@f-graphene were also found to be durable and extremely stable as explained above. Hence it can be concluded that the RhNPs immobilized on the functionalized graphene support is an ideal and better candidate that can substitute the platinum electrode (Platinum on porous carbon support) in fuel cells.

## Conclusion

In summary, a series of transition metal nanoparticles (RhNPs, PtNPs, CoNPs, FeNPs) and their composites with f-graphene (RhNPs@f-graphene, PtNPs@f-graphene, CoNPs@f-graphene) have been prepared and characterized by SEM, TEM and p-XRD analytical methods. The nanoparticles were mostly aggregated and segregate upon composite formation with f-graphene. The RhNPs immobilized on f-graphene resemble the dispersion of water droplets on a lotus leaf. The RhNPs and the composite RhNPs@f-graphene electrocatalyze hydrogen evolution from acidic water at much lower potentials than that of PtNPs and PtNPs@f-graphene under identical experimental conditions. The reduction onset potentials for RhNPs and RhNPs@f-graphene were found to be E_p_, −0.117 V and −0.186 V respectively whereas for the PtNPs and PtNPs@f-graphene were E_p_, −0.367 V and −0.38 V respectively. The CoNPs, FeNPs and CoNPs@f-graphene electrocatalyze hydrogen evolution at higher reduction potentials E_p_, −0.97 V, −1.58 V and −0.98 V respectively. The lower electropotentials and high current values observed in the case of RhNPs and RhNPs@-graphene reveal the crucial role played by rhodium in electrocatalytic hydrogen evolution. The plausible mechanism could be the formation of hydridic Rh-H^−^ species under the influence of electrode potential which interact with further proton to form hydrogen gas.

## Methods

### Preparation of RhNPs, PtNPs, CoNPs and FeNPs

The metal halides, RhCl_3_.3H_3_O or anhydrous PtCl_2_ or CoCl_2_.6H_2_O or anhydrous FeCl_3_ (0.05 g) was stirred in distilled deionized water (30 mL) at 30 °C for 5 min. NaBH_4_ (0.02 g) was added to the reaction mixture in portions carefully. A bright red colored solution was obtained in the case of RhCl_3_.6H_2_O which became colorless after the precipitation of black particles. After stirring for 8 h, the black precipitate was collected by filtration. The precipitate was washed with water (10 mL), sonicated with acetone (25 mL), centrifuged and the acetone layer was decanted. The black precipitate thus obtained was dried and weighed (0.02 g; yield, >95%).

### Preparation of functionalized grapheme

Graphite powder (0.5 g) was taken in THF (80 mL) and water (20 mL), stirred at 38 °C for 2 h and ultra-sonicated for 10 h. The solvents were decanted after centrifugation and the residue was dried thoroughly. This residue was treated carefully and dropwise with concentrated H_2_SO_4_ (30 mL) and fuming nitric acid (10 mL) at 0 °C. The reaction mixture was heated under reflux for 12 h and allowed to stand at 38 °C for 10 h. The supernatant acid layer was decanted and the residue was washed thoroughly with water by centrifugation and dried. FT-IR (*v*, cm^−1^): 3400 (br), 1714 (br.), 1600 (w).

### Preparation of the Composite, TMNPs@graphene

RhNPs/PtNPs/CoNPs (0.02 g) was mixed with functionalized graphene (0.02 g) in degassed LC-MS grade CH_3_CN (12 mL) and ultra-sonicated in closed sample vials under argon atmosphere for 3 h. The reaction mixtures were then evaporated to dryness and the residues were used in catalytic experiments.

## Supplementary information

ESI
