# Supplementary material for: Graphene Supported Rhodium Nanoparticles for Enhanced Electrocatalytic Hydrogen Evolution Reaction
Source: Sci Rep. 2019 Nov 19;9:17027. doi: 10.1038/s41598-019-53501-x (PMC6863816; doi:10.1038/s41598-019-53501-x)
Supplement: Supplementary file 1 — ESI [file 41598_2019_53501_MOESM1_ESM.docx]

**Electronic Supplementary Information**

**Graphene Supported Rhodium Nanoparticles for Enhanced Electrocatalytic Hydrogen Evolution Reaction**

*Ameerunisha Begum^1*^, Moumita Bose^2^ and Golam Moula^2^*

^1^Department of Chemistry, Faculty of Science, Jamia Hamdard University, New Delhi – 110062, India. E-mail: abegum@jamiahamdard.ac.in

^2^Department of Chemistry, University of Calcutta, Acharya Prafulla Chandra Road, Calcutta – 700009, West Bengal, India.

**Contents**

- 1. Materials & Methods
  2. Cyclic voltammograms of CoNPs@f-graphene as a function of increasing concentrations of added p-TsOH.
  3. Cyclic voltammograms of PtNPs@f-graphene as a function of increasing concentrations of added p-TsOH.
  4. TEM image of the RhNPs after the cyclic voltammetric experiment.
  5. SEM image of the RhNPs after the cyclic voltammetric experiment
  6. TEM image of the recycled RhNPS.
  7. SEM image of CoNPs.

1. **Materials and methods**

All solvents and chemicals were purchased from commercial sources. Functionalized graphene was prepared as described previously.^[28]^ Scanning electron microscopic measurements were done by coating the samples on aluminium stubs and scanned using the SUPRA 40VP Field Emission Scanning Electron Microscope (CARL ZEISS NTS GmbH, Oberkochen (Germany) equipped with energy dispersive X-ray (EDX) facility. TEM measurements were done by coating the samples on to polymer film coated copper grids and scanned using the FEI Technai 20 U Twin Transmission Electron Microscope equipped with EDS detector. Cyclic voltammetric studies were carried out at 298 K on a BASi Epsilon-Eclipse Bioanalytical systems in H_2_O. Glassy carbon working, platinum wire auxiliary and Ag/AgCl with 3.0 M KCl reference electrodes were used. Potassium nitrate of 0.2 M concentration was used as supporting electrolyte in water. Gas chromatographic analysis of H_2_ gas was performed on Agilent Technologies 7890A GC system using N_2_ carrier gas. A mixture of RhNPs@graphene (0.05 g) and TsOH (0.8g, ~4 mmol) in water (KNO_3_ supporting electrolyte, 0.2M) was purged with nitrogen for 15 min. A 2 ml syringe was inserted into the compartment of electrochemical cell and the argon gas supply inlet was closed. The reaction mixture was subjected to controlled potential electrolysis at -0.6 V, the gas bubbles formed at the GC working electrode surface were tapped off to the surface and the gas over the solution was taken into the syringe which was then injected into the GC system.

1. **Cyclic voltammograms of CoNPs@f-graphene as a function of increasing concentrations of added p-TsOH.**

**Fig. S1.** Cyclic voltammograms of CoNPs@f-graphene as a function of increasing concentrations of added p-TsOH in water. scan rate of 100 mVs^–1^ (Supporting electrolyte, KNO_3_/H_2_O (0.2 M), GCE working, Pt wire auxillary and Ag/AgCl reference electrodes)

1. **Cyclic voltammograms of PtNPs@f-graphene as a function of increasing concentrations of added p-TsOH**

**Fig. S2.** Cyclic voltammograms of PtNPs@f-graphene as a function of increasing concentrations of added p-TsOH in water. scan rate of 100 mVs^–1^ (Supporting electrolyte, KNO_3_/H_2_O (0.2 M), GCE working, Pt wire auxillary and Ag/AgCl reference electrodes)

1. **TEM image of the RhNPs after the cyclic voltammetric experiment**





**Fig. S3.** TEM image of the RhNPs after the cyclic voltammetric experiment displaying presumably KNO3 and p-toluene sulfonate crystals.

1. **SEM image of the RhNPs after the cyclic voltammetric experiment**

**Fig. S4.** SEM image of the RhNPs after the cyclic voltammetric experiment displaying presumably KNO_3_ and p-toluene sulfonate crystals.

1. **TEM image of the recycled RhNPS.**





**Fig. S5.** TEM image of the recycled RhNPS.

1. **SEM image of CoNPs**

**Fig. S6.** SEM image of CoNPs.
